# Potency of moderate-intensity exercise on caspase-8 and caspase-9 expression in a DMBA-induced oral epithelial dysplasia rat model

**DOI:** 10.1590/1678-7765-2026-0438

**Published:** 2026-08-24

**Authors:** Anis Irmawati, Rasha Fatimah Abdullah, Athallah Reyhan Darmawan, Retno Indrawati Roestamadji, Ira Arundina, Noor Faizah Balqis, Fiki Muhammad Ridho, Noeroel Widajati, Raed Labib

**Affiliations:** 1 Universitas Airlangga Faculty of Dental Medicine Department of Basic Dental Science and Public Health Surabaya Indonesia Universitas Airlangga, Faculty of Dental Medicine, Department of Basic Dental Science and Public Health, Surabaya, Indonesia.; 2 Universitas Airlangga Faculty of Dental Medicine Surabaya Indonesia Universitas Airlangga, Faculty of Dental Medicine, Profession Program, Surabaya, Indonesia.; 3 Universitas Airlangga Faculty of Public Health Magister Program of Epidemiology Surabaya Indonesia Universitas Airlangga, Faculty of Public Health, Magister Program of Epidemiology, Surabaya, Indonesia.; 4 Universitas Airlangga Faculty of Dental Medicine Surabaya Indonesia Universitas Airlangga, Faculty of Dental Medicine, Postgraduate Student, Surabaya, Indonesia.; 5 Department of Occupational Health and Safety Faculty of Public Health Universitas Airlangga Surabaya Indonesia Department of Occupational Health and Safety, Faculty of Public Health, Universitas Airlangga, Surabaya, Indonesia.; 6 University of Medical and Applied Sciences Faculty of Dental Medicine Department of Oral Surgery Sana’a Yemen University of Medical and Applied Sciences, Faculty of Dental Medicine, Department of Oral Surgery, Sana’a, Yemen.

**Keywords:** Moderate-intensity exercise, Caspase-8, Caspase-9, Oral dysplasia, DMBA, Good health and well-being

## Abstract

**Background:**

Oral squamous cell carcinoma (OSCC) accounts for approximately 90% of oral cancers, with tobacco exposure representing one of its major risk factors. The tobacco carcinogen 7,12-dimethylbenz(a)anthracene (DMBA) induces oral epithelial dysplasia, a premalignant lesion. Caspase-8 and caspase-9 are initiator caspases that trigger apoptosis in dysplastic cells. Currently, disease management focuses on prevention. Moderate-intensity exercise has been reported to modulate apoptosis-related signaling and may contribute to cancer prevention.

**Objective:**

To evaluate the potency of moderate-intensity exercise on caspase-8 and -9 expression in a DMBA-induced oral dysplasia rat model.

**Methodology:**

A total of 24 Wistar rats were randomly divided into four groups: K1 (no exercise, no DMBA), K2 (exercise, no DMBA), K3 (no exercise, DMBA), K4 (exercise, DMBA). Exercise consisted of moderate-intensity swimming at 70% MWC, performed 3x/week for 6 weeks. DMBA (0.02 mg/kg body weight) was injected into the buccal mucosa of the canine region twice weekly for 2 weeks. At the 7^th^ week, the rats were anesthetized with ketamine (10 mg/kg body weight), euthanized by cervical dislocation, and buccal tissue specimens were collected. Immunohistochemical staining was performed to obtain caspase-8 and -9 expression. Data were analyzed in SPSS version 29.0.

**Results:**

All groups presented significant differences in caspase-8 (*p* = 0.004) and caspase-9 (*p* <0.001) expression. Caspase-8 and caspase-9 expression was significantly higher in K4 than in K3, with expression restored to levels comparable to the untreated control group (K1).

**Conclusion:**

Moderate-intensity exercise restored caspase-8 and caspase-9 expression suppressed by DMBA exposure, suggesting preservation of apoptotic signaling in DMBA-induced Wistar rats.

## Introduction

Cancer is a major public health problem worldwide, being a leading cause of death in both developed and developing countries.^1^ In 2022, approximately 19.98 million new cancer cases were reported globally, of which 9.74 million resulted in death, whereas Indonesia recorded approximately 6,515 new oral cancer cases and over 3,500 deaths.^2^ In several South Asian countries, including India, Sri Lanka, Pakistan, and Bangladesh, oral cancer accounted for 33% of all reported cancers.^3^

Oral cancer often begins with clinical signs in the mouth known as precancerous lesions—e.g., oral erythroplakia, stippled leukoplakia, and lichen planus—which can be prevented by early identification. However, these lesions are often overlooked because they are painless, resulting in 70% of oral cancer cases being diagnosed at a more advanced stage, requiring complex, expensive, and lengthy treatment and carrying a poor prognosis. Research conducted at Dharmais Cancer Hospital between 2003 and 2013 found that the life expectancy of oral cancer patients in Indonesia is only 24 months, worse than other common types of cancer in the country. In developing countries, over 50% of oral cancers originate from precancerous lesions.^4^

Oral cancer typically appears on the lips, buccal mucosa, tongue, gums, nasopharynx, larynx, and oropharynx.^5,6^ Of all oral cancer cases worldwide, 90% are oral squamous cell carcinoma (OSCC), making it the most common malignancy of the oral cavity.^7^ This disease is often diagnosed at an advanced stage, leading to low life expectancy, especially in populations with high-risk behaviors such as smoking, betel nut chewing, and alcohol consumption.^8^ According to a questionnaire study, over 40.3% of respondents in Indonesia reported being active smokers, and 89% of these smokers were male. This percentage exceeds the global figure.^9^

High smoking prevalence places a significant burden on public health due to its toxic substances, one of which is tar. Tar contains polycyclic aromatic hydrocarbons, including 7,12-dimethylbenz(a)anthracene (DMBA).^10^ Experimental studies have shown that OSCC can be reliably induced in animal models using DMBA, which promotes carcinogenesis by initiating DNA damage.^11^ Chronic exposure of the oral epithelium to DMBA induces a series of histopathological changes, including hyperkeratosis, epithelial hyperplasia, and dysplasia, ultimately leading to the development of well-differentiated OSCC. These lesions closely resemble the morphological and molecular features observed in human OSCC.^12^

Oral epithelial dysplasia (OED) is a condition of the oral mucosa identified via histological examination of structural and cytological alterations in oral epithelium. Genomic mutations often lead to OED development and its transformation into OSCC. Although some OED cases are caused by human papillomavirus infection, most cases are caused by chemical carcinogens such as tobacco and alcohol. As OED represents a reversible stage before malignant transformation, intervention targeting this stage may provide an effective strategy for oral cancer prevention.^4,13^

Current approaches to managing OSCC include surgery, radiotherapy, chemotherapy, targeted therapy, and immunotherapy. Recent developments in targeted therapy show promise as a significant improvement in oral cancer treatment. These treatments focus on specific molecular pathways involved in cancer growth, potentially resulting in more effective and less harmful options. Immunotherapy offers a new way to treat oral cancer by using immune system to fight cancer cells. Recent advances in immunotherapy have yielded encouraging results in clinical studies.^14^

Regular physical activity has been associated with protection against various non-communicable diseases.^15^ However, evidence regarding its role in preventing oral carcinogenesis remains limited and controversial. Thus, this study evaluated the potency of moderate-intensity exercise on caspase-8 and caspase-9 expression in a DMBA-induced oral dysplasia rat model.

## Methodology

### Study design and ethical approval

This research was a purely experimental study with a post-test only control group design. Ethical eligibility has been reviewed and approved by the Health Research Ethics Committee of the Faculty of Dental Medicine, Universitas Airlangga, Surabaya, Indonesia, with certificate no. 0828/HRECC.FODM/VIII/2025.

### Experimental animals

Experimental animals were healthy male Wistar rats (*Rattus norvegicus),* aged 1–2 months and weighing 100–250 grams, obtained from Farma Veterinary Center, Directorate General of Livestock and Animal Health Ministry of Agriculture, Surabaya, Indonesia. All animals were clinically healthy upon arrival and showed no signs of infection or physical abnormalities. Inclusion criteria consisted of: 1) healthy and active white male Wistar rats; 2) body weight between 100–250 grams, appropriate for their age; 3) aged between 1-2 months; and 4) normal physical anatomy. Exclusion criteria included: 1) rats that appeared sick or inactive (passive) before intervention; 2) deformed rats; and 3) rats that died during the experiment.

Sample size for each group was calculated using Lemeshow. Based on the calculation formula, the minimum number of replicates per group obtained was four rats. To minimize the loss of experimental units, a correction test was applied with estimated dropout proportion of 25%. The calculated value of corrected sample size was 5.33, which when rounded up required 6 Wistar rats per group for observation. Thus, the total sample size required was 24 Wistar rats.

Animals were housed in plastic cages lined with rice husk bedding in the cage section at Biochemistry Laboratory, Faculty of Medicine, Universitas Airlangga, Surabaya, Indonesia. Each cage housed four rats in air-conditioned room maintained at approximately 20°C under a 12-hour light/12-hour dark cycle. Rats were fed a commercial diet (HI-PRO-VITE 511B; crude protein 20–23%, minimum crude fat 5%, supplemented with calcium and phosphorus) once daily at 12:00 PM (20 g/rat/day). Drinking water was provided *ad libitum*.

Animals were acclimatized for 2 weeks before the intervention under the 3Rs (Replacement, Reduction, Refinement) principles and the Five Freedoms (freedom from hunger and thirst; from environmental discomfort such as temperature, humidity, and appropriate housing; from pain, injury, and diseases; from fear and distress; to express normal behavior).

### Materials and equipment

Tools used consisted of: a rat cage (30 x 40 x 25 cm); analytical scale for weighing the rat’s body weight and load; a water tank used as a rat swimming pool, with capacity of 150 liters, 62 cm in diameter, and 83 cm in height; metal for weights; a set of surgical instruments (surgical scissors, scalpel, needle set no. 15) and an operating table; stopwatch; box for storing tissue, labeling equipment, markers, disposable syringes, microtome, incubator, stirrer, measuring cup, centrifuge, tissue paper, gloves, masks, object glass, pint, nierbekken, cover glass, microscope.

Materials used included DMBA, distilled water, ketamine hydrochloride, xylol, alcohol, 10% formalin buffer, 10% EDTA, paraffin, phosphate buffered saline (PBS), corn oil, peroxidase inhibitor solution, hematoxylin, diaminobenzidine (DAB) chromogen, diluted inhibitory serum, and anti-caspase-8 antibody (clone EPR162, Cat. No. ab108333, Abcam, Cambridge, UK) and anti-caspase-9 antibody (clone EPR18868, Cat. No. ab184786, Abcam, Cambridge, UK).

### DMBA preparation

The DMBA solution was prepared at the Research Center, Faculty of Dental Medicine, Universitas Airlangga, Surabaya, Indonesia, by dissolving 1 mg of DMBA powder in 1 mL of corn oil and mixing it until homogeneous using a vortex.

### Experimental grouping and intervention

All rats were randomly divided into four groups using simple random sampling: K1 (no exercise, no DMBA), K2 (exercise, no DMBA), K3 (no exercise, DMBA), and K4 (exercise and DMBA). No missing data were observed during the experiment. Rats in the non-DMBA groups (K1 and K2) were handled on the same schedule as the DMBA-treated groups, but did not receive sham or vehicle injections.

Before the intervention, the animals underwent a preliminary exhaustive swimming test to determine their individual maximum work capacity. Four rats swam simultaneously in a cylindrical water tank used as a swimming pool (62 cm in diameter and 83 cm in height) containing approximately 150 L of room-temperature water. Each rat was continuously monitored throughout the swimming session. Exhaustion was defined as the inability to maintain coordinated swimming movements and to keep the nose above water, with failure to return to the surface within approximately five seconds. At this point, the rat was immediately removed from the water. Swimming duration was then multiplied by 70% to determine the exercise duration for moderate-intensity exercise.

Exercise intervention consisted of swimming with an additional load equivalent to 3% of body weight, which was tied to 1/3 of the base of the rat’s tail. Four rats swam simultaneously during each exercise session, performing moderate-intensity swimming at 70% of their individual maximum work capacity, three times per week for six weeks.

A DMBA solution was intramuscularly injected into the buccal mucosa of the right upper jaw in the canine region at a dose of 0.02 mg/kg body weight and a depth of 2-3 mm. Injection volume was adjusted according to each animal’s body weight while maintaining a constant DMBA concentration. Injections were performed twice weekly for 2 weeks during the 5^th^ and 6^th^weeks, while maintaining the same exercise treatment.

### Tissue collection

At the 7^th^ week, all DMBA-treated rats (groups K3 and K4) developed clinically visible nodular lesions. These nodular lesions were surgically excised for histopathological and immunohistochemical analyses. Animals were anesthetized using 10 mg/kg body weight intramuscular ketamine hydrochloride. Deep anesthesia was confirmed by the absence of withdrawal response to forceps pinch before tissue excision. While still unconscious, the rats were euthanized by cervical dislocation. Death was confirmed by the absence of spontaneous respiration. The euthanized rats were then cleaned, wrapped in cloth, and buried to a depth of approximately 25–50 cm. The excised tissues were then fixed in 10% buffered formalin for at least 48 hours, then processed for paraffin block preparation in the Anatomical Pathology Laboratory, Faculty of Medicine, Universitas Airlangga, Surabaya, Indonesia.

After fixation, tissue dehydration was performed using graded alcohol, namely a concentration of 70% for 15 minutes, 80% for 1 hour, 90% for 2 hours, 95% for 1 hour, and 100% for 1 hour, repeated thrice. Subsequently, the tissue was immersed in xylol clarifying agent thrice, with a duration of 1 hour, 2 hours and 2 hours. The next impregnation process was by immersing the tissue wrapped in filter paper and labeled in paraffin at a temperature of 56–60 °C, for 24 hours. After paraffin hardening, the paraffin block was sectioned using a microtome with a thickness of 4-5 µm and the sections were placed on glass slides. Representative paraffin sections were stained with standard hematoxylin and eosin. Histopathological examination was performed by an experienced oral and maxillofacial pathologist to confirm the presence of epithelial dysplasia in all DMBA-injected rats prior to immunohistochemical analysis.

### Immunohistochemistry staining

Immunohistochemistry (IHC) staining was performed at the Biomolecular Biochemistry Laboratory, Faculty of Medicine, Universitas Brawijaya, Malang, Indonesia. Paraffin sections were deparaffinized in xylol twice for 5 minutes each and rehydrated with 100% (2x), 90% (2x), and 70% ethanol for 3 minutes each, followed by immersing in water for 1 minute. Heat-induced epitope retrieval (HIER) was performed using a Decloaking Chamber before blocking endogenous peroxidase activity. Endogenous peroxidase activity was blocked using a peroxidase inhibitor solution at 37 °C for 10 minutes, followed by incubation in prediluted blocking serum at temperature of 25 °C for 10 minutes. The slides were immersed in anti-caspase-8 and anti-caspase-9 antibody at a dilution of 1:1000 for 10 minutes at 25 °C. After rinsing with phosphate-buffered saline for 5 minutes, the sections were incubated with secondary antibody and peroxidase, receiving PBS washes between each step. Immunoreactivity was visualized using DAB chromogen, counterstained with hematoxylin for 3 minutes, rinsed under running water, and covered with a cover glass.

### Outcome assessment

Caspase-8 and caspase-9 expression was observed under a light microscope at 400x magnification. For each rat, five microscopic fields were randomly selected from the dysplastic oral epithelium of each tissue section. Positive cells were identified by brown staining and counted as positive caspase-8 or caspase-9 cells. The observer responsible for microscopic evaluation was blinded to the experimental group allocation. Tissue slides were coded before assessment, and group identities were disclosed only after all measurements had been completed. Data were expressed in mean and standard deviation (SD).

### Statistical analysis

Primary outcomes consisted of caspase-8 and caspase-9 expression. Data were analyzed on SPSS version 29.0 (IBM Corp., Armonk, NY, USA). Normality was assessed by Shapiro-Wilk testing, and data homogeneity was evaluated using Levene’s test. Differences between groups were analyzed using one-way ANOVA. Pairwise comparisons used Fisher’s protected LSD post hoc test only after a statistically significant ANOVA result was obtained. Statistical significance was set at *p* <0.05.

## RESULTS

All animals (n = 6 rats/group) completed the study; no animals died, were excluded, or had unusable tissue sections. All DMBA-treated rats (groups K3 and K4) developed clinically visible nodular lesions characterized by localized swelling and erythema at the injection site (**Figure 1**). No unexpected procedure-related complications or adverse events were observed during the experimental period.

**Figure 1:**
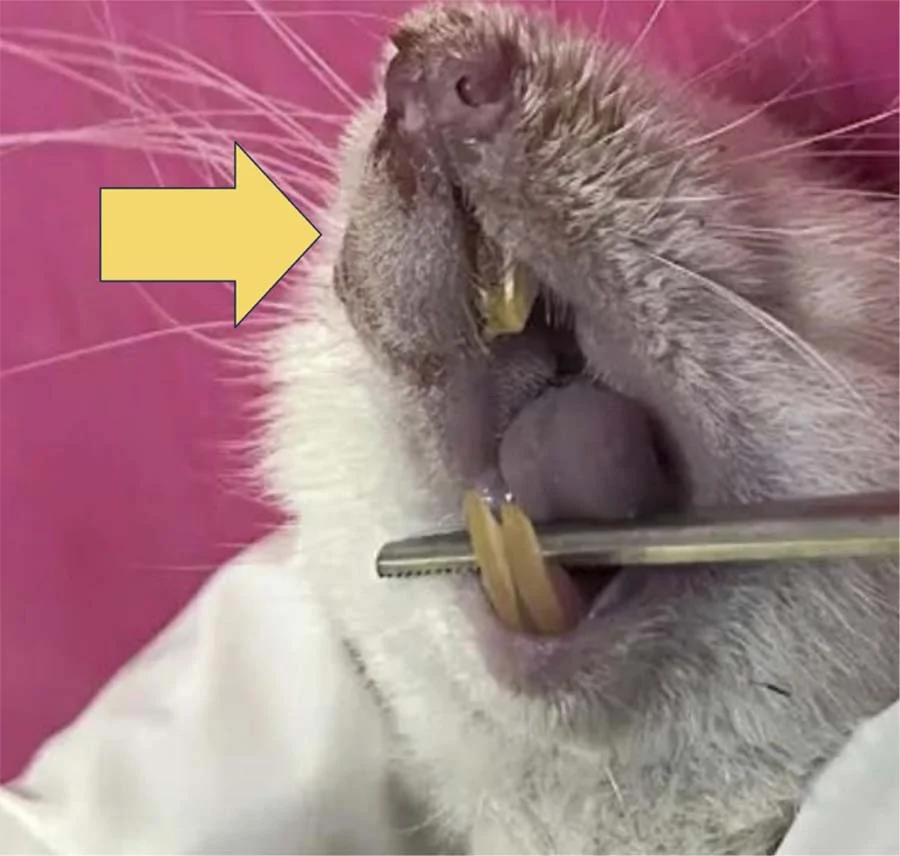
Clinical appearance of the upper right buccal mucosal tissue of the rat oral cavity after DMBA induction. Swelling and erythema were observed.

Parametric testing assumptions were evaluated. The Shapiro-Wilk test showed that caspase-8 and caspase-9 expression data were normally distributed in all groups (*p* >0.05). Levene’s test indicated homogeneity of variances among groups for both outcomes (*p* >0.05).

In the DMBA-induced oral dysplasia model, moderate-intensity exercise restored the expression of both caspase-8 and caspase-9 compared with the DMBA-only group. Caspase-8 expression was higher in K4 than in K3 (3.67 ± 0.816 vs. 2.50 ± 0.548), whereas caspase-9 expression was also higher in K4 than in K3 (3.83 ± 0.753 vs. 2.33 ± 0.516), reaching levels comparable to those observed in the control group (K1) (**Figure 2**).

**Figure 2:**
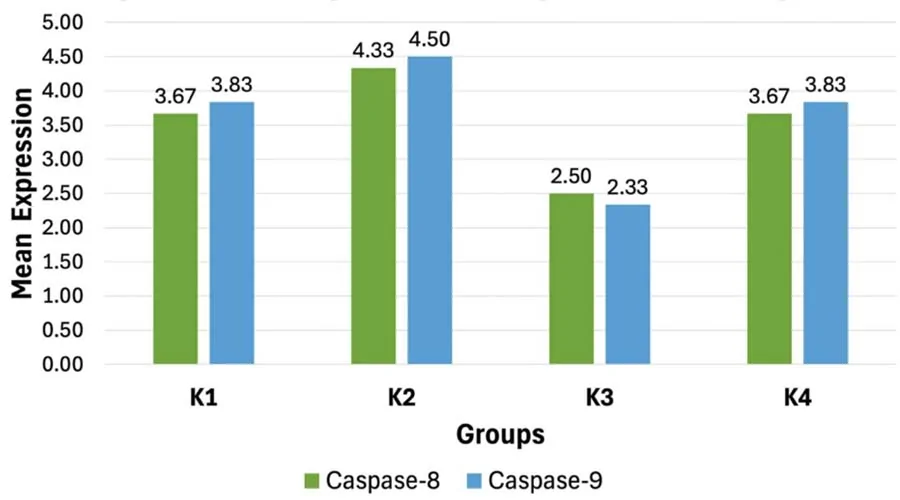
Expression of caspase-8 and caspase-9 in four groups.

One-way ANOVA test yielded significant overall differences among the four groups for both caspase-8 (*p* = 0.004) and caspase-9 (*p* <0.001) (**Table 1**).

**Table 1:** Comparison of caspase-8 and caspase-9 expression among groups using one-way ANOVA.

Group	*n*	Caspase-8		Caspase-9	
		Mean ± SD	*p*-value	Mean ± SD	*p*-value
K1	6	3.67 ± 0.816	0.004*	3.83 ± 0.753	<0.001*
K2	6	4.33 ± 0.816		4.50 ± 0.837	
K3	6	2.50 ± 0.548		2.33 ± 0.516	
K4	6	3.67 ± 0.816		3.83 ± 0.753	

* Significant difference.

Pairwise comparisons using Fisher’s LSD test confirmed that caspase-8 (*p* = 0.015) and caspase-9 (*p* = 0.002) expression were significantly higher in the DMBA plus exercise group (K4) than in the DMBA-only group (K3), indicating that moderate-intensity exercise attenuated the DMBA-associated reduction in caspase expression. Additional significant differences were also observed between K1 and K3 (caspase-8: *p* = 0.015; caspase-9: *p* = 0.002), and between K2 and K3 (caspase-8: *p* <0.001; caspase-9: *p* <0.001) (**Table 2**). **Figure 3** and **Figure 4** presents the representative IHC staining of caspase-8 and -9 in the four groups, respectively.

**Table 2:** Pairwise comparisons of caspase-8 and caspase-9 expression with Fisher’s LSD post hoc test.

Group	K1	K2	K3	K4
**K1**		0.143^a^	0.015^a,^*	1.000^a^
		0.127^b^	0.002^b,^*	1.000^b^
**K2**	0.143^a^		<0.001^a,^*	0.143^a^
	0.127^b^		<0.001^b,^*	0.127^b^
**K3**	0.015^a,^*	<0.001^a,^*		0.015^a,^*
	0.002^b,^*	<0.001^b,^*		0.002^b,^*
**K4**	1.000^a^	0.143^a^	0.015^a,^*	
	1.000^b^	0.127^b^	0.002^b,^*	

* Significant difference. ^a^ Caspase-8. ^b^ Caspase-9.

**Figure 3:**
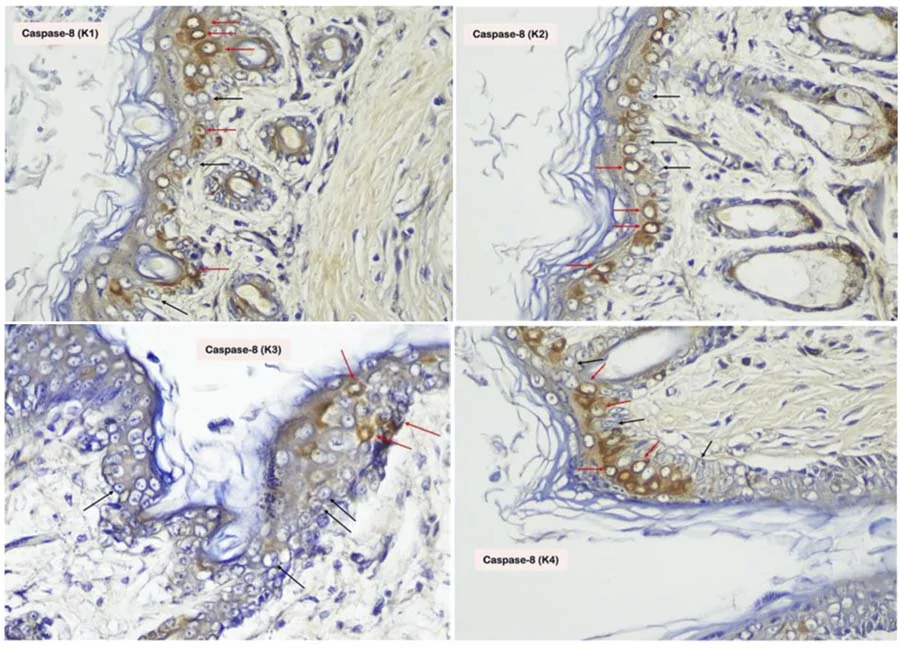
Expression of caspase-8 in all groups. The expression was observed under a light microscope at 400x magnification. Red arrows indicate positive cells.

**Figure 4:**
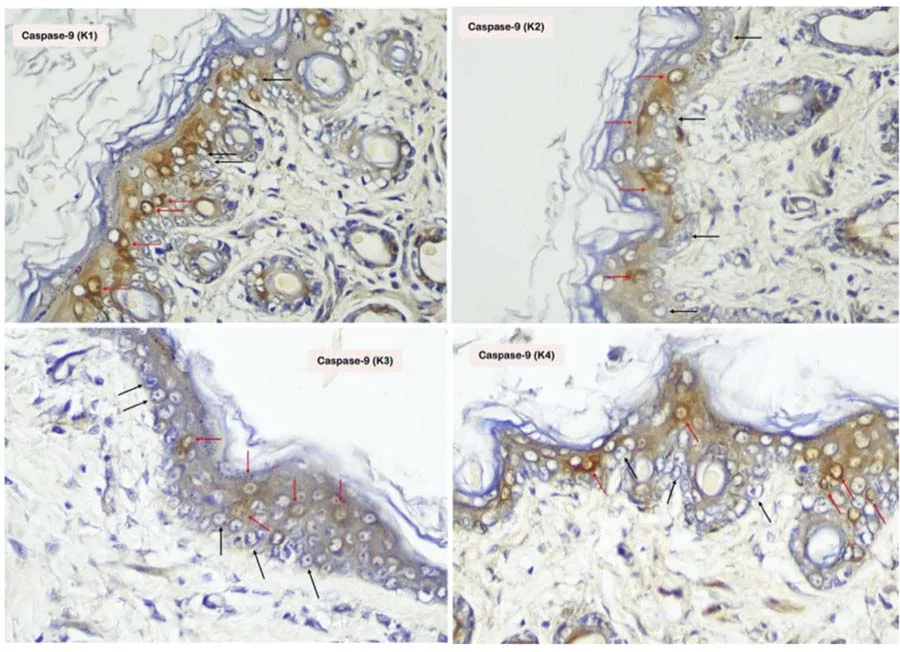
Expression of caspase-9 in all groups. The expression was observed under a light microscope at 400x magnification. Red arrows indicate positive cells.

## DISCUSSION

This study showed that moderate-intensity exercise significantly restored caspase-8 expression in DMBA-induced oral dysplasia. Caspase-8 expression was significantly higher in the exercise-treated DMBA group (K4) than in the DMBA-only group (K3), whereas its expression returned to a level comparable with the untreated control group (K1). Together with the similar findings observed for caspase-9, these results suggest that moderate-intensity exercise may preserve the apoptotic signaling that is suppressed by DMBA exposure rather than increasing apoptosis beyond physiological levels. However, these findings should be interpreted cautiously since the control groups did not receive sham or vehicle injections, which may have influenced caspase-8 and caspase-9 expression. Additionally, as epithelial dysplasia severity was not histopathologically graded, the potential influence of differences in dysplasia severity on caspase expression between the experimental groups cannot be excluded.

Caspase-8 was selected as the research focus because it is a key initiator of the extrinsic apoptosis pathway and serves as a molecular bridge between the extrinsic and intrinsic pathways through its ability to cleave BID to tBID.^16^ This dual function makes caspase-8 a strategic target for cancer prevention. Moreover, although several studies have examined executioner caspases and intrinsic pathway regulators such as Bcl-2 and Bax, relatively limited research has investigated the specific modulation of caspase-8 expression through lifestyle interventions such as exercise, particularly in oral cancer prevention contexts.^17^ Understanding caspase-8 regulation is crucial because its suppression has been identified as a key mechanism in cancers, including OSCC, allowing tumor cells to evade apoptosis despite accumulating genetic damage.^18^

Interestingly, the exercise-only group (K2) showed numerically higher caspase-8 expression than the untreated control group (K1). However, no statistically significant difference was observed between these groups; thus, the present findings do not support the conclusion that moderate-intensity exercise increases apoptotic activity in normal oral epithelium. Instead, this observation may indicate a physiological adaptation that maintains cellular homeostasis without inducing excessive apoptosis.^7,19^

One possible mechanism for the caspase-8 expression restoration observed here is the modulation of apoptosis-related signaling pathways previously described in experimental studies. Moderate-intensity exercise has been reported to induce transient intracellular Ca^2^ influx, leading to activation of Ras-GAP and Src and mitogen-activated protein kinase (MAPK) signaling which subsequently enhances p53 activity. Increased p53 activity may promote Fas receptor expression and facilitate activation of the extrinsic apoptotic pathway through caspase-8.^7,19^ Conversely, the marked reduction of caspase-8 expression observed in the DMBA-only group may reflect the suppressive effects of DMBA-induced oxidative stress and chronic inflammation. Previous studies have suggested that DMBA activates DAMP-TLR4-NF-κB signaling, resulting in sustained inflammatory responses, impaired p53 activity, reduced Fas expression, and increased anti-apoptotic signaling through cytokines and Bcl-2.^19,20^ These provide biologically plausible mechanisms that may explain the restoration of caspase-8 expression following moderate-intensity exercise.

The caspase-8 expression restoration observed may also be related to the ability of moderate-intensity exercise to maintain redox homeostasis. A key factor in this protective mechanism is the regulation of reactive oxygen species (ROS). They function not only as potentially harmful oxidants but also as essential signaling molecules involved in cell homeostasis, immune function, and apoptosis regulation.^21,22^ Moderate-intensity exercise achieves optimal ROS balance through hormesis, in which moderate stress activates cellular defense systems following a bell-shaped curve pattern.^23^ Based on the hormesis mechanism, Mi, et al.^24^ (2019) showed that 16-week moderate-intensity exercise enhanced endogenous antioxidant defenses by increasing Sirtuin-3 (SIRT3) and Superoxide Dismutase 2 (SOD2). Collectively, the previous findings suggest that improved redox balance may partly explain the preservation of apoptotic signaling observed after moderate-intensity exercise.

Conversely, high-intensity or exhaustive exercise produces excessive ROS, which paradoxically creates an anti-apoptotic environment that is detrimental to cancer prevention. When ROS production exceeds cellular antioxidant capacity, cells interpret this as a threat to survival and activate defense mechanisms that protect damaged cells from apoptosis.^25^ This excessive oxidative stress overwhelms cellular antioxidant defenses, leading to chronic inflammation and activation of pro-survival pathways, including NF-κB, which in turn up regulates Bcl-2.^20^ Moreover, these studies have shown that exhaustive exercise can accumulate large amounts of ROS, which can directly damage DNA and potentially promote cancer development and growth rather than prevent it. This creates a dangerous scenario where damaged cells that would otherwise be eliminated through apoptosis are instead protected and allowed to survive, potentially progressing to malignancy. Overall, these findings suggest that moderate-intensity exercise may provide a more favorable biological environment than high intensity exercise for maintaining apoptotic homeostasis.^20,25^

Regarding caspase-9, this study evinced that expression was significantly higher in the DMBA plus exercise (K4) than in the DMBA-only group (K3). Similar to the caspase-8 findings, DMBA exposure alone was associated with reduced caspase-9 expression, whereas moderate-intensity exercise restored its expression to a level comparable with the untreated control group (K1). These findings suggest that moderate-intensity exercise may preserve intrinsic apoptotic signaling suppressed by DMBA rather than enhancing apoptosis beyond physiological levels.

Moderate-intensity exercise may counteract the pro-survival environment induced by DMBA. Previous studies have shown that DMBA-induced oxidative stress may activate DAMP-TLR4-NF-κB signaling, resulting in sustained inflammatory responses, increased anti-apoptotic proteins such as Bcl-2, reduced Bax expression, and suppression of the intrinsic apoptotic pathway.^19,20,26^ Consequently, reduced caspase-9 expression in the DMBA-only group may reflect impaired mitochondrial apoptosis under chronic inflammatory conditions.

Previous studies suggest that moderate-intensity exercise may promote intrinsic apoptosis by activating Ca^2^-dependent signaling pathways that enhance p53 activity, thereby improving the balance between pro-apoptotic and anti-apoptotic proteins, including Bcl-2 and Bax.^27,28^ A favorable Bax/Bcl-2 balance facilitates mitochondrial outer membrane permeabilization, cytochrome c release, apoptosome formation, and subsequent activation of caspase-9, ultimately initiating the downstream apoptotic cascade.^29,30^ These pathways may explain caspase-9 expression restoration following moderate-intensity exercise in the present study.

In addition to direct cellular effects, previous studies reported that exercise may enhance NK cell activity, improve immune surveillance, and reduce chronic inflammation through modulation of pro- and anti-inflammatory cytokines.^31^ Consistent with these observations, Irmawati, et al.^32^ (2026) reported that moderate-intensity swimming reduced salivary IL-8 and TNF-α levels in young male smokers. Additionally, Zhu, et al.^33^ (2022) showed that exercise slowed cancer progression in preclinical models and improved immune parameters in clinical studies. Collectively, these findings support the concept that the beneficial effects of moderate-intensity exercise extend beyond individual tissues and may contribute to systemic biological adaptations associated with cancer prevention.

Several study limitations should be acknowledged. First, the non-DMBA groups did not receive sham or vehicle injections; thus, the potential influence of local injection procedures cannot be completely excluded. Second, although all DMBA-treated rats developed clinically visible nodular lesions and epithelial dysplasia was histopathologically confirmed, dysplasia severity was not graded and lesion size was not quantitatively assessed; thus, variations in the severity of dysplastic lesions may have contributed to the observed differences in caspase-8 and caspase-9 expression among the experimental groups. Third, this study evaluated only the immunohistochemical expression of caspase-8 and caspase-9 as indicators of apoptosis-related signaling. As immunohistochemistry reflects protein expression rather than functional activation of apoptosis, the present findings should be interpreted as evidence of altered expression of apoptosis-related proteins rather than direct evidence of apoptosis. Moreover, other molecular pathways involved in apoptosis and carcinogenesis were not directly measured. Future studies should incorporate appropriate sham controls, standardized dysplasia grading, and comprehensive molecular and functional assays, such as TUNEL staining, cleaved caspase immunostaining, or caspase activity assays, to further elucidate the mechanism by which moderate-intensity exercise modulates apoptosis during oral carcinogenesis.

## Conclusion

Moderate-intensity exercise restored caspase-8 and caspase-9 expression suppressed by DMBA exposure, indicating preservation of apoptotic signaling during oral epithelial dysplasia. These findings suggest that moderate-intensity exercise may help counteract carcinogen-associated dysregulation of apoptosis and support its potential as a complementary strategy for oral cancer prevention. Further studies incorporating comprehensive molecular and functional analyses are needed to elucidate the underlying mechanisms and evaluate their translational relevance.
